# Decitabine/Cedazuridine in the Management of Myelodysplastic Syndrome and Chronic Myelomonocytic Leukemia in Canada

**DOI:** 10.3390/curroncol30090581

**Published:** 2023-08-30

**Authors:** John Paul Yun, Philip Q. Ding, Aastha Dolley, Winson Y. Cheung

**Affiliations:** 1 Oncology Outcomes Program, Department of Oncology, University of Calgary, Calgary, AB T2N 1N4, Canada; johnpaul.yun@ucalgary.ca (J.P.Y.); philip.ding@albertahealthservices.ca (P.Q.D.); 2 Galway University Hospital, H91 YR71 Galway, Ireland; 3 Faculty of Medicine & Dentistry, University of Alberta, Edmonton, AB T6G 2R3, Canada; 4 Taiho Pharma Canada, Inc., Oakville, ON L6H 5R7, Canada; adolley@taihopharma.ca; 5 Department of Oncology, Cumming School of Medicine, University of Calgary, Calgary, AB T2N 1N4, Canada

**Keywords:** myelodysplastic syndrome, chronic myelomonocytic leukemia, real-world evidence, hypomethylating agents, treatment patterns, patient support programs

## Abstract

The management of myelodysplastic syndrome (MDS) and chronic myelomonocytic leukemia (CMML) is limited and remains an unmet need. Decitabine/cedazuridine (DEC-C, ASTX727) is Canada’s first and only approved oral hypomethylating agent for MDS and CMML. We characterized the real-world use of DEC-C through a Canadian compassionate use program. Demographic and clinical data from 769 patients enrolled in Taiho Pharma Canada’s Patient Support Program were collected and analyzed. These patients represent a collection period from 10 November 2020 to 31 August 2022 with a median age of 76 years. Among 651 patients who started DEC-C, the median treatment duration was 4.2 cycles. The median overall and progression-free survival were 21.6 and 10.7 months, respectively. Among 427 patients who discontinued treatment, the majority (69.5%) stopped due to death (*n* = 164) or disease progression (*n* = 133). Multivariable cox regression showed that age, province of residence, blast counts, antibiotic prophylaxis, and number of dose reductions and delays were not significantly associated with overall and progression-free survival. DEC-C is a promising alternative to parenteral hypomethylating agent therapy, and it likely addresses an important unmet need for effective and convenient therapies in this setting.

## 1. Introduction

Myelodysplastic syndrome (MDS) encompasses a heterogeneous group of hematopoietic stem cell disorders characterized by ineffective hematopoiesis and dysplastic changes in myeloid, erythroid, and megakaryocytic progenitors [1]. The resulting pancytopenia and increased risk of progression to acute myeloid leukemia (AML) cause significant morbidity and mortality in patients with MDS [2]. Chronic myelomonocytic leukemia (CMML) is an overlapping myelodysplastic/myeloproliferative malignancy [3]. In Canada, the estimated age-standardized incidence of MDS is 3.69 per 100,000 [4]. CMML is less common, with an average incidence rate of 2.45 cases per million individuals annually [5]. While long-term epidemiological studies in Canada remain limited, yearly incidence rates have increased in several countries including Germany, the UK, and the United States [6]. In combination with increasing physician awareness and improved diagnostic classifications, it is likely that MDS in Canada’s aging population will demonstrate similar trends in years to come [4].

With a median age of diagnosis between 70 and 76 years [2,4,7], most MDS patients are elderly with a varied prognosis depending on disease-related factors [8]. Patients with MDS may be classified according to the International Prognostic Scoring System (IPSS), by degree of pre-leukemic blast expansion, response to therapeutic agents, disease outcomes, and prognosis [9]. Based on the IPSS score, patients can be further classified as “lower risk MDS” (LR-MDS), which includes IPSS low risk or intermediate-1, and comprises approximately 70% of patients with a median survival of 3.5–5.7 years. “Higher risk MDS” (HR-MDS) includes IPSS intermediate-2 and high-risk categories with a median survival of 0.4–1.2 years [9]. Newer IPSS-R and IPSS-M classification updates include both cytogenetic and molecular features not collected in this study [10].

LR-MDS is often treated with hematopoietic growth factors, transfusions, and other supportive care measures. HR-MDS is typically treated with hypomethylating agents (HMAs) with the minority of patients undergoing intensive chemotherapy and hematopoietic stem cell transplantation (HSCT) [11]. The management of complications in MDS is critical as more than 80% of patients are anemic (hemoglobin < 100 g/L) at diagnosis and more than 50% of patients become red blood cell transfusion dependent during the course of their disease [12,13]. HSCT remains the only potentially curative therapy for MDS and CMML patients and is generally reserved for patients without significant comorbidity due to a high risk of complications [3,14,15,16]. For patients who are not candidates for HSCT, HMAs are an effective treatment option for those with HR-MDS and as appropriate, LR-MDS [11]. Azacitidine and decitabine are the cytidine analogues most commonly used as HMAs. These single-agent therapies were approved by Health Canada for the treatment of patients with HR-MDS (and CMML for decitabine) who are not candidates for HSCT [17,18,19,20]. Although these drugs have been shown to induce hematologic improvement in approximately one-third of patients, their demanding subcutaneous (SC)/intravenous (IV) infusion schedules prove to be a barrier for treatment continuation [21]. While a Canadian national guideline for the management of MDS remains unavailable, Cancer Care Ontario guidelines acknowledge intravenous HMA’s potential role in transfusion dependence reversal and its use in compassionate palliative settings [22].

Decitabine/cedazuridine (DEC-C, ASTX727, INQOVI^®^) is an oral fixed-dose combination tablet containing 35 mg of decitabine and 100 mg of cedazuridine. DEC-C was developed by Astex Pharmaceuticals and is distributed by Taiho Pharma Canada. Cedazuridine is an inhibitor of cytidine deaminase in the gut and liver that increases the systemic exposure of decitabine. DEC-C’s recommended dose is one tablet once daily on days 1–5 of each 28-day cycle. It was approved by Health Canada on July 7, 2020 for the treatment of adult patients diagnosed with MDS and CMML based (IPSS intermediate-1, intermediate-2, and high risk) or CMML [23].

DEC-C demonstrated similar pharmacokinetic, pharmacodynamic, and safety profiles of IV decitabine in a 2019 phase 1 study [24]. Subsequent phase 2 and phase 3 studies evaluated daily oral dosing regiments of 5 days in each 28-day cycle. The median treatment duration in the phase 2 study reported seven cycles (range 1–29) with a median follow up of 24.3 months. Of the eighty patients, forty-eight (60%) reported clinical responses including 17 (21%) with complete responses. Of those with baseline red blood cell transfusion dependence (*n* = 38), 50% (*n* = 19) became transfusion independent. Half of the 12 patients with baseline platelet transfusion dependence achieved transfusion independence at the end of Phase 2 studies [25]. In the ASCERTAIN phase 3 study, the median number of cycles was 9 and median follow-up was 32 months. In addition, 22% of patients achieved complete response, 26% proceeded to HSCT, and 53% became transfusion-independent for both red blood cells and platelets. Safety findings in both phase 2 and 3 studies were consistent with those anticipated for IV-DEC (related Grade ≥ 3 AEs in more than 5% were thrombocytopenia, neutropenia, anemia, febrile neutropenia, and leukopenia) [26]. Currently, the cost of DEC-C in Canada is comparable to and in some cases lower than generic azacytidine, and all Canadian provinces and territories except for Quebec now fund DEC-C according to the product label.

To our knowledge, the real-world use of oral DEC-C for intermediate-1 to high-risk MDS and CMML in Canada has yet to be studied since approval in 2020. Using data from Canadians enrolled to receive DEC-C through Taiho Pharma Canada’s Patient Support Program (PSP), we characterized demographic and clinical parameters of this patient cohort. As a secondary objective, we assessed both safety and tolerability, treatment duration, and survival outcomes alongside factors that may impact each of the above.

## 2. Materials and Methods

### 2.1. Study Design and Population

This was a retrospective cohort study of patients from Canadian provinces receiving oral DEC-C through Taiho Pharma Canada’s PSP. All patients included for analysis were adults (≥18 years of age) diagnosed with IPSS intermediate-1, intermediate-2, or high risk MDS (previously treated or untreated, de novo or secondary) or CMML, and who received treatment with oral DEC-C through the PSP. Patients in our analysis also reported an Eastern Cooperative Oncology Group (ECOG) performance status of 2 or less. Approval for this study was obtained from the Health Research Ethics Board of Alberta Cancer Committee.

### 2.2. Study Data

Study data were collected by Bayshore HealthCare and provided by Taiho Pharma Canada. Demographical and clinical characteristics were collected during patient enrollment, including age at diagnosis, province of residence, and IPSS risk score. Treatment characteristics included enrollment date, treatment status, reason for status, treatment start date, treatment stop date, reimbursement information, and any treatment modification if applicable. At the 6th cycle of oral DEC-C therapy, physicians were sent a re-enrollment form to complete. This form assisted in collecting updated patient characteristics including treatment status, red blood cell and platelet transfusion status, maintenance of blast counts, use of prophylactic antibiotics, and any dose reductions or delays in the last 6 cycles. Our analysis reflects a data collection period from 10 November 2020 to 31 August 2022.

### 2.3. Statistical Analysis

Descriptive statistics were used to summarize baseline patient and treatment characteristics. Continuous variables were presented as medians with interquartile ranges and means with standard deviations while categorical variables were presented as frequencies and percentages. Subgroup analysis was performed to analyze potential relationships between clinical parameters. Patients were grouped as LR-MDS (IPSS status intermediate-1) or HR-MDS (IPSS status intermediate-2 and high risk) as per IPSS [9]. Further analysis explored parameters based on treatment duration. Subgroups were divided between patients receiving greater than, or equal to, 4 cycles of DEC-C versus less than 4 cycles. Each cycle is defined in 28-day periods as per the product monograph [22]. The 6th cycle re-enrollment data were also utilized to compare the transfusion status against initial enrollment forms to note any change in the last 6 cycles.

Progression-free survival (PFS) and overall survival (OS) were estimated using the Kaplan–Meier method and utilizing time-to-event data involving date of enrollment to date of event (i.e., treatment discontinuation due to death or disease progression).

Wilcoxon rank-sum tests were used for continuous variables while Pearson’s Chi-square test or Fischer’s exact test were used for categorical variables. Multivariable Cox regression analysis was performed using likelihood ratio tests to find associations between survival outcomes and select demographic/clinical factors. The significance level of all statistical two-sided tests was defined a priori as <0.05. All analyses were performed using R Studio [27].

## 3. Results

Across Canadian provinces, 769 patients were enrolled in the Taiho Pharma Canada PSP to receive oral DEC-C for the treatment of MDS and CMML. The median age at enrollment was 76 years (range 21–97 years). Examining patient enrollment by geographical location, the greatest number of patients resided in Ontario (*n* = 357, 46.4%). Patients from Quebec reflected the second largest proportion of patients (*n* = 150, 19.5%). The remaining patients were distributed across Western provinces (British Columbia, Alberta, Saskatchewan, Manitoba) (*n* = 178, 23.1%) and Atlantic provinces (New Brunswick, Newfoundland and Labrador, Nova Scotia, Prince Edward Island) (*n* = 84, 10.9%). Of patients with recorded IPSS risk scores, the greatest proportion of patients (40.9%, *n* = 277) had intermediate-1 risk MDS. Patients with intermediate-2 risk totaled 212 (31.3%) while 177 patients (26.1%) had high risk of disease. Of patients with reported transfusion dependence status at enrollment, 60.2% were red blood cell transfusion dependent while 16.1% of patients were platelet transfusion dependent. Six hundred and fifty-one patients (84.7%) started treatment on oral DEC-C with a median time from enrollment to treatment start of 12 days (range 0–273). Median treatment duration for patients who discontinued therapy was 4.2 cycles (range 0.0–22.7). The majority of patients (*n* = 570, 74.7%) were reimbursed by a compassionate use program funded by Taiho Pharma Canada (Table 1).

**Figure 1:**
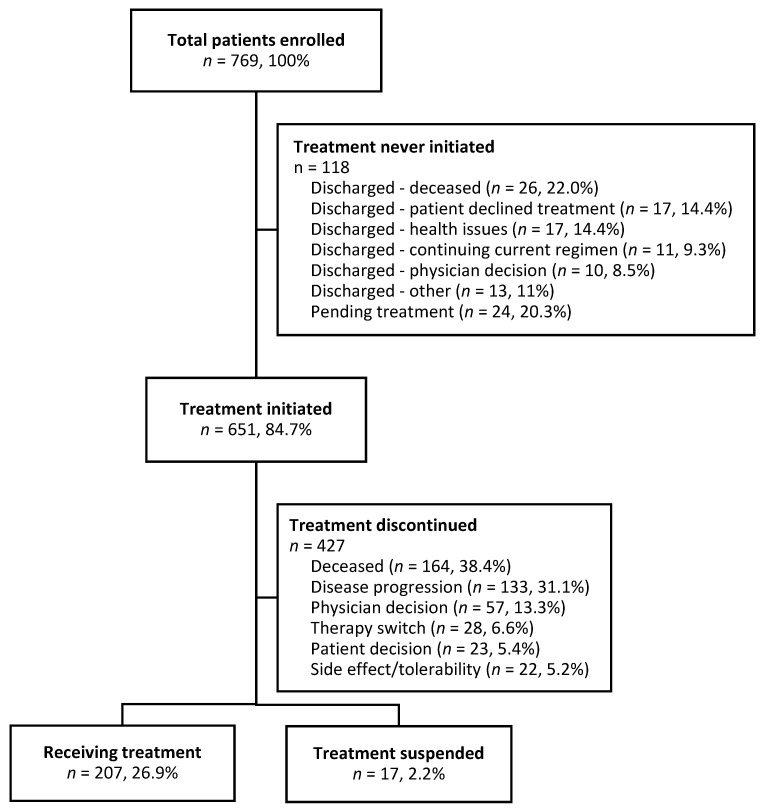
Flow diagram of patients enrolled to receive DEC-C.

Of 769 patients enrolled, 118 (15.3%) never started DEC-C during the data collection period. The vast majority (79.7%) of these patients were discharged from the program for reasons outlined in Figure 1, while others (*n* = 24, 20.3%) were pending treatment initiation. Of the 651 patients who initiated treatment, 427 patients discontinued treatment during the study period with the most common reasons being death (38.4%), disease progression (33.1%), and physician decision (13.3%). Two hundred and seven patients (26.9%) were still on treatment at the data collection cut-off date (Figure 1).

Stratifying patients by IPSS risk group, there were 389 patients with HR-MDS and 277 with LR-MDS. While there were more patients who died in the higher-risk subgroup (*n* = 97, 44.3%) versus the lower-risk subgroup (*n* = 49, 32.0%), this is expected, and the difference was not statistically significant. There were no statistically significant differences in treatment duration or red blood cell/platelet transfusion dependence status at enrollment between the HR-MDS and LR-MDS subgroups (Table 2). Of 155 patients who received the 6th cycle re-enrollment form, data from 120 (77%) patients were collected. Of these patients, 108 patients reported both IPSS score and the number of dose reductions in the last six cycles. Fifty-eight patients (53.7%) did not require any dose reduction. Comparing IPSS risk groups, a greater proportion of patients with HR-MDS did not require any dose reduction in the last six cycles compared to those with LR-MDS (60.3% vs. 42.5%, *p* = 0.03). Eighty-eight patients had both IPSS status and red blood cell transfusion status reported at the 6th cycle. The greatest proportion of patients had maintained red blood cell transfusion independence since enrollment. Of 90 patients with both IPSS scores and platelet transfusion status reported at the 6th cycle, the greatest proportion had maintained platelet transfusion independence since enrollment. Both antibiotic prophylaxis status and IPSS status was reported in 108 patients during the re-enrollment process among which only eight patients (7.8%) received antibiotic prophylaxis in the last 6 cycles (Table 2).

Subgroup analyses based on treatment duration revealed that a significantly greater proportion of patients who received <4 cycles stopped treatment due to death, patient decision, or side effect compared to those who received ≥4 cycles (*p* < 0.001). Conversely, treatment discontinuation due to disease progression, physician decision, or therapy switch was more common in patients who received ≥ 4 cycles. No significant differences between treatment duration subgroups were shown for age, province, or time to treatment initiation (Table 3).

**Figure 2:**
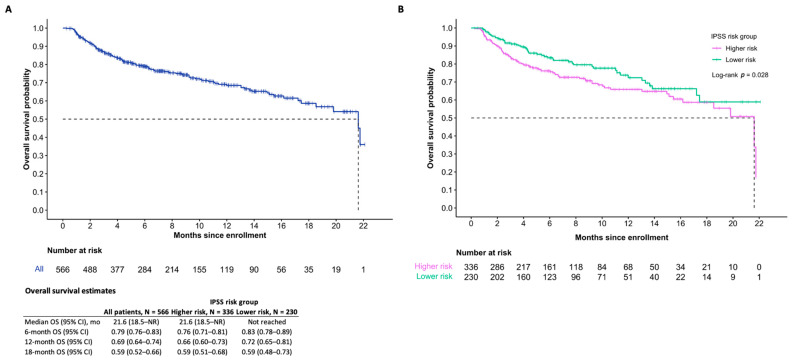
Overall survival of patients (**A**) and IPSS risk subgroups (**B**) of those who initiated treatment.

**Figure 3:**
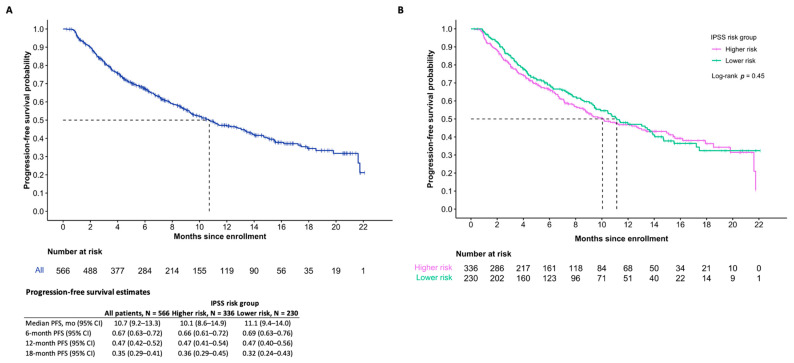
Progression-free survival (**A**) and IPSS risk subgroups (**B**) of those who initiated treatment.

At the time of the data cut-off, the median OS among all patients who received DEC-C was 21.6 months (95% confidence interval (CI) 18.5-inf). The median PFS overall was 10.7 months (95% CI 9.2–13.3). Differences in OS between IPSS risk groups were statistically significant (*p* = 0.03), while differences in PFS were not statistically significant (*p* = 0.45). Survival estimates are further summarized in Figure 2 and Figure 3.

Multivariable cox regression analysis revealed no significant associations between OS, PFS and various demographic and clinical factors (i.e., patient age at enrollment, patient province, IPSS risk group, time to treatment initiation, blast count status, antibiotic prophylaxis, dose reductions, and dose delays) (Table 4).

## 4. Discussion

This study examines real-world oral DEC-C use for the treatment of MDS and CMML through the Taiho Pharma Canada PSP. To our knowledge, the use of DEC-C in the Canadian real-world setting has yet to be described in the literature since its approval by Health Canada. There were 769 patients who enrolled into the PSP during our study period, which represents a notable uptake of this novel oral therapeutic. Interest and preference toward oral formulations over IV/SC chemotherapy is well documented in the literature. Indeed, a recent 2022 study analyzing the online survey data from MDS patients revealed both a preference and perceived personal benefit from improved quality of life from receiving oral DEC-C in comparison to IV/SC treatment [21]. The distribution of IPSS risk subgroups within patients with MDS in our cohort was similar to that of representative patient samples in the existing literature, where the greatest proportion of patients was INT-1 followed by INT-2 and then high risk [14,26]. The median age of our cohort was 76 years old (Range: 21–97). This is similar to the Canadian median age of diagnosis of 75 years for both MDS and CMML [4,5], and it is consistent with epidemiological studies characterizing both MDS and CMML to be diseases of older age [28,29]. The median age of our cohort was approximately five years older than that of previous clinical trials exploring oral DEC-C for MDS and CMML [24,25,26]. This difference may be explained by the more extensive eligibility criteria that trials use, which builds study cohorts that may not represent the diverse population of patients for whom DEC-C may be prescribed in the real world.

The median OS of patients who received oral DEC-C was 21.6 months. This was significantly less than the median OS in the phase 3 study ASCERTAIN which demonstrated a median OS of 31.7 months [30]. A large proportion of patients in our study stopped treatment due to death (*n* = 164, 38.4%) or disease progression (*n* = 133, 33.1%). In comparison, the ASCERTAIN phase 3 study reported treatment discontinuation for disease progression in only six patients (4.5%) while reporting no deaths while on treatment. These discrepancies may be explained by ASCERTAIN’s more stringent enrollment criteria which included eligibility for IV decitabine administration, life expectancy of three or more months, adequate organ function, ECOG performance status of 0–1, and fewer than 1 prior cycle of azacitidine or decitabine [31]. As such, patients enrolled in the phase 3 ASCERTAIN study were likely healthier at baseline and at lower risk of earlier death or disease progression compared to the present study cohort, which comprised all for whom DEC-C was indicated. Additionally, the data collection period reflects a unique timeframe in society where the COVID-19 pandemic brought unprecedented challenges to both the individual patient and healthcare systems at large. MDS patients, by the nature of their mean age and disease, were more susceptible to downstream effects of the virus. From treatment delays, scarcity of transfusion products, to death from COVID-19 virus itself, MDS patients have been well described in the literature to have suffered multiple negative consequences [32,33]. Future studies may reveal the true extent of COVID-19 on survival outcomes data during this time.

Our study revealed a median treatment duration of 4.2 cycles (range 0.0–22.7) for patients who received therapy and similar durations between LR- and HR-MDS. This median duration is in keeping with one other study exploring real-world data of DEC-C in the United States [34]. However, the median treatment duration for patients in ASCERTAIN was significantly longer at nine cycles [30]. Research has demonstrated that the longer treatment duration of HMA is associated with improved clinical outcomes, including higher reported response rates, reduced AML transformation, and improved OS [35,36,37,38,39,40]. Specifically, clinical trial evidence has suggested that patients require four to six cycles of HMA therapy to achieve a clinical response [36,41,42,43]. However, prior real-world data suggest many patients do not persist with HMA treatment after initiation, receiving less than four or six cycles or having gaps over 90 days between cycles [41]. This is consistent with Canadian data from the national MDS Registry, with one study finding 33% of patients with HR-MDS receive <4 cycles of subcutaneous hypomethylation therapy with short overall survival [44]. Delayed response to hypomethylation therapy is not unusual; hence, for patients with stable disease or those who achieve clinical response from HMA, the continuation of therapy remains closely associated with improved survival [45]. This is reflected in current guidelines from the National Comprehensive Cancer Network (NCCN) and the European Society for Medical Oncology (ESMO) [14,15]. Consistent with the evidence that longer treatment duration is associated with superior survival outcomes, our study found that a significantly greater proportion of patients who received <4 cycles stopped treatment due to death versus those who received ≥4 cycles (49.3% vs. 28.4%, *p* < 0.001).

Reasons for early HMA discontinuation can be clinical in nature (e.g., patient mortality, disease progression, adverse effects) and/or non-clinical (e.g., logistical challenges, provider inexperience, socioeconomic barriers). In this study, oral DEC-C is shown to be a well-tolerated drug with 5.2% of patients (*n* = 22) stopping therapy due to side effects/tolerability issues. DEC-C has a tolerability profile consistent with IV decitabine [46] but is not impacted by the complex logistics inherent with IV HMA treatment which may result in early discontinuation or dose delay [47]. Of the 119 patients in this study who were followed up at 6 cycles, 55 patients (46.2%) reported no dose delays in their therapy, 30 patients (25.2%) received 1 dose delay, 14 patients (11.8%) received 2 dose delays, while 20 patients (16.8%) received 3 or more dose delays. Although most patients experienced one dose delay or less, reasons for dose delay may have been of a clinical or non-clinical nature and will require further investigation.

Transfusion dependence is not uncommon among MDS and CMML patients [12]. Approximately 50–90% of patients will require red blood cell transfusions with 30–50% requiring ≥1 platelet transfusion [48]. Most patients (60.2%) in our study were red blood cell transfusion dependent at enrollment with some (16.1%) who were platelet transfusion dependent. Transfusion dependence is associated with poorer quality of life and survival outcomes in MDS. As HMAs have been shown to promote transfusion independence in both LR-MDS [49] and HR-MDS [50], reporting any change in transfusion status is valuable in assessing the real-world performance of DEC-C. Of patients receiving ≥6 cycles of DEC-C, 19.8% achieved a change in transfusion status from being red blood cell transfusion dependent at enrollment to independent at the 6th cycle versus 9.3% who shifted from red blood cell transfusion independent to dependent. Red blood cell transfusion independence was maintained in 40.7% of patients after 6 cycles.

The cytopenic complications of MDS and CMML and the associated predisposition to infections warrant the consideration of antibiotic prophylaxis. Current guidelines recommend prophylactic antibiotics use for patients at high risk of febrile neutropenia, profound protracted neutropenia, and patients on hematopoietic stem cell therapy [51]. Additional recommendations include anti-viral and anti-fungal coverage in specific clinical scenarios. In this study, 57.0% of patients were reported as maintaining baseline blast counts from enrollment to cycle 6. This is important to quantify, as hypomethylating agents may cause transient neutropenia especially during initial cycles of therapy and increasing risks of infection [35,37,52,53]. Multivariable cox regression models revealed no statistically significant impact of blast count on OS or PFS. During DEC-C treatment, 8.7% reported being on prophylactic antibiotics.

This study contains inherent limitations which should be considered. Due to the nature of data collection and necessary privacy, we were not permitted to link other data sources; thus, we lacked access to data points not captured within the PSP. These may include prior treatment history, leukemic transformation prior to therapy, and specifics on antibiotic prophylaxis. Another limitation was the inability to identify specific reasons for patients’ decisions not to receive DEC-C nor the rationales for physician decisions to stop DEC-C. Parsing these decisions in future studies may allow for more thorough assessments of non-persistence due to logistical factors, patient preferences, or provider inexperience. Furthermore, while the PSP was effective in facilitating early access to oral DEC-C to Canadians, this dataset may not be fully representative in the setting of wider access involving public drug funding plans and private reimbursement. Finally, due to the retrospective nature of our study analysis, the associations explored in our analysis are susceptible to unmeasured confounding variables.

Our study highlights the role that PSP and compassionate use programs serve in providing access and financial support to Canadians who require novel therapeutics like HMAs. Most patients in this study (64.4%, *n* = 496) were reimbursed through Taiho Pharma Canada’s compassionate support program. By enabling early patient access to oral DEC-C in the context of MDS and CMML, the program facilitates foundational research which may allow for more comprehensive decision making regarding public drug funding. Additionally, despite data supporting the use of HMAs in the treatment of HR-MDS and CMML, several studies have suggested that approximately half of patients with HR-MDS do not receive HMA therapy at all [41,53,54,55,56,57]. As real-world data on oral DEC-C’s performance in the management of MDS and CMML are limited, the PSP enables timely research to help address this knowledge gap and optimize HMA use.

## Figures and Tables

**Table 1:** Demographic and clinical characteristics of the patient cohort.

Characteristic	*N*	Overall, *N* = 769
Age at enrollment, years	769	
Mean ± standard deviation (SD)		75 ± 10
Median (Range)		76 (21–97)
<75		337 (43.8%)
≥75		432 (56.2%)
Patient province	769	
Ontario		357 (46.4%)
Western provinces		178 (23.1%)
Quebec		150 (19.5%)
Atlantic provinces		84 (10.9%)
International Prognostic Scoring System (IPSS) risk score at enrollment	678	
Intermediate-1		277 (40.9%)
Intermediate-2		212 (31.3%)
High		177 (26.1%)
N/A (CMML)		12 (1.8%)
Time to treatment initiation, days	651	
Mean ± SD		19 ± 25
Median (Range)		12 (0–273)
Red blood cell transfusion dependent at enrollment	598	360 (60.2%)
Platelet transfusion dependent at enrollment	598	96 (16.1%)
Treatment duration, cycles	427	
Mean ± SD		5.5 ± 4.5
Median (Range)		4.2 (0.0–22.7)
<4		205 (48.0%)
≥4		222 (52.0%)
Reimbursement type	763	
Compassionate		570 (74.7%)
Bridging		82 (10.7%)
Private		82 (10.7%)
Public		28 (3.7%)
Cash Paying		1 (0.1%)

**Table 2:** (**a**) Patient characteristics by IPSS risk group, overall. (**b**) Patient characteristics by IPSS risk group, 6th cycle follow-up.

(a)					
Characteristic	*N*	Overall, *N* = 666	IPSS Risk Group		*p* Value
			Higher Risk, *N* = 389	Lower Risk, *N* = 277	
Age at enrollment, years	666				0.54
Mean ± SD		75 ± 10	75 ± 9	74 ± 11	
Median (Range)		76 (21–97)	76 (27–97)	75 (21–96)	
Treatment status	666				0.65
Treatment discontinued		372 (55.9%)	219 (56.3%)	153 (55.2%)	
Receiving treatment		180 (27.0%)	108 (27.8%)	72 (26.0%)	
Never started treatment		100 (15.0%)	53 (13.6%)	47 (17.0%)	
Treatment suspended		14 (2.1%)	9 (2.3%)	5 (1.8%)	
Reason for treatment discontinuation	372				0.11
Death		146 (39.2%)	97 (44.3%)	49 (32.0%)	
Disease progression		120 (32.3%)	61 (27.9%)	59 (38.6%)	
Physician decision		45 (12.1%)	26 (11.9%)	19 (12.4%)	
Therapy switch		25 (6.7%)	13 (5.9%)	12 (7.8%)	
Patient decision		18 (4.8%)	13 (5.9%)	5 (3.3%)	
Side effect/tolerability		18 (4.8%)	9 (4.1%)	9 (5.9%)	
Treatment duration, cycles	372				0.19
Mean ± SD		5.5 ± 4.5	5.3 ± 4.6	5.6 ± 4.3	
Median (Range)		4.2 (0.0–22.7)	4.1 (0.1–22.7)	4.3 (0.0–18.0)	
Red blood cell transfusion dependent at enrollment	549	333 (60.7%)	194 (59.3%)	139 (62.6%)	0.44
Platelet transfusion dependent at enrollment	547	89 (16.3%)	47 (14.5%)	42 (18.9%)	0.17
**(b)**					
**Characteristic**	**N**	**Overall, ** ***N* = 108**	**IPSS Risk Group**		***p* Value**
			**Higher Risk,** ***N* = 68**	**Lower Risk, ** ***N* = 40**	
Red blood cell transfusion dependent at 6 cycles	88	32 (36.4%)	21 (39.6%)	11 (31.4%)	0.43
Change in red blood cell transfusion dependence from enrollment to 6th cycle	82				0.66
TI to TI *		34 (41.5%)	20 (39.2%)	14 (45.2%)	
TD to TI **		16 (19.5%)	10 (19.6%)	6 (19.4%)	
TD to TD		25 (30.5%)	15 (29.4%)	10 (32.3%)	
TI to TD		7 (8.5%)	6 (11.8%)	1 (3.2%)	
Platelet transfusion dependent at 6 cycles	90	13 (14.4%)	8 (14.3%)	5 (14.7%)	0.96
Change in platelet transfusion dependence from enrollment to 6th cycle	86				1.00
TI to TI *		69 (80.2%)	45 (80.4%)	24 (80.0%)	
TD to TI **		5 (5.8%)	3 (5.4%)	2 (6.7%)	
TD to TD		5 (5.8%)	3 (5.4%)	2 (6.7%)	
TI to TD		7 (8.1%%)	5 (8.9%)	2 (6.7%)	
Blast count maintained	85	49 (57.6%)	26 (51.0%)	23 (67.6%)	0.13
Antibiotic prophylaxis use	103	8 (7.8%)	6 (9.2%)	2 (5.3%)	0.71
Dose reductions	108				**0.03**
0		58 (53.7%)	41 (60.3%)	17 (42.5%)	
1		33 (30.6%)	21 (30.9%)	12 (30.0%)	
2+		17 (15.7%)	6 (8.8%)	11 (27.5%)	
Dose delays	108				0.372
0		50 (46.3%)	30 (44.1%)	20 (50.0%)	
1		30 (27.8%)	22 (32.4%)	8 (20.0%)	
2–3		28 (25.9%)	16 (23.5%)	12 (30.0%)	

* TI: Transfusion Independence, ** TD: Transfusion Dependence, *p* values in bold are statistically significant.

**Table 3:** Baseline demographics and treatment characteristics by treatment duration, patients who initiated and discontinued treatment only.

Characteristic	*N*	Overall, *N* = 427	Treatment Duration, Cycles		*p* Value
			<4, *N* = 205	≥4, *N* = 222	
Age at enrollment, years	427				0.21
Mean ± SD		74 ± 10	75 ± 10	74 ± 10	
Median (Range)		75 (21–97)	76 (39–97)	75 (21–95)	
Patient province	427				0.11
Ontario		187 (43.8%)	79 (38.5%)	108 (48.6%)	
Western provinces		97 (22.7%)	52 (25.4%)	45 (20.3%)	
Quebec		92 (21.5%)	44 (21.5%)	48 (21.6%)	
Atlantic provinces		51 (11.9%)	30 (14.6%)	21 (9.5%)	
Reason for treatment discontinuation	427				**<0.001**
Death		164 (38.4%)	101 (49.3%)	63 (28.4%)	
Disease progression		133 (31.1%)	56 (27.3%)	77 (34.7%)	
Physician decision		57 (13.3%)	10 (4.9%)	47 (21.2%)	
Therapy switch		28 (6.6%)	7 (3.4%)	21 (9.5%)	
Patient decision		23 (5.4%)	13 (6.3%)	10 (4.5%)	
Side effect/tolerability		22 (5.2%)	18 (8.8%)	4 (1.8%)	
Time to treatment initiation, days	427				0.27
Mean ± SD		19 ± 24	18 ± 22	20 ± 25	
Median (Range)		12 (0–233)	12 (0–233)	13 (2–218)	

*p* values in bold are statistically significant.

**Table 4:** Multivariable cox regression models, patients who initiated treatment only.

Characteristic	Overall Survival		Progression-Free Survival	
	HR (95% CI)	*p* Value	HR (95% CI)	*p* Value
Age at enrollment, years		0.74		1.00
<75	Reference		Reference	
≥75	0.78 (0.19–3.23)		1.00 (0.33–3.07)	
Patient province		0.78		0.63
Ontario	Reference		Reference	
Western provinces	1.60 (0.18–13.95)		0.94 (0.21–4.17)	
Quebec	2.66 (0.41–17.45)		0.89 (0.24–3.36)	
Atlantic provinces	1.56 (0.12–20.57)		0.28 (0.03–2.50)	
IPSS risk group		1.00		0.92
Higher risk	Reference		Reference	
Lower risk	1.00 (0.24–4.18)		1.05 (0.38–2.94)	
Time to treatment initiation	1.02 (0.96–1.09)	0.47	1.02 (0.97–1.06)	0.43
Blast count maintained		0.94		0.90
No	Reference		Reference	
Yes	0.94 (0.21–4.22)		0.93 (0.31–2.81)	
Antibiotic prophylaxis use		0.97		0.89
No	Reference		Reference	
Yes	1.04 (0.10–10.59)		0.86 (0.09–7.74)	
Dose reductions		0.71		0.86
0	Reference		Reference	
1	1.33 (0.24–7.38)		1.02 (0.30–3.52)	
2+	2.59 (0.26–26.08)		0.70 (0.16–3.01)	
Dose delays		0.74		0.42
0	Reference		Reference	
1	0.86 (0.13–5.75)		1.69 (0.42–6.76)	
2+	0.44 (0.05–3.95)		2.44 (0.65–9.19)	

## Data Availability

Data will not be shared due to patient confidentiality, according to ethics approval for this study.
